# Starting study of microbial genetics diversity in soils of mangrove preserved in sergipe

**DOI:** 10.1186/1753-6561-8-S4-P189

**Published:** 2014-10-01

**Authors:** Karla Cristina Santos Freire, Francine Ferreira Padilha, Caroline Olinda, Denize Oliveira

**Affiliations:** 1 Doutoranda do Programa de Doutorado em Biotecnologia, RENORBIO, Fortaleza, CE, Brazil; 2 Doutora e Pesquisadora do Programa de Doutorado em Biotecnologia, RENORBIO, Fortaleza, CE, Brazil; 3 Graduanda em Biomedicina, Universidade Tiradentes, Aracaju, SE, Brazil

## 

Tropical mangroves are considered one of the most productive ecosystems in the world, characterized by high rate of cycling of organic matter and nutrients that occurs between the oceans and terrestrial environments. This paper proposes the characterization and evaluation of structures and diversity of microbial communities in the soil of mangroves in three conservation areas located in Sergipe. The samples were analyzed by the extraction, purification and analysis molecucar. Soil samples were collected in PVC tubes 50 cm long and 50 mm in diameter, placed vertically in the sediment of three different regions of the state of Sergipe, in triplicate. For the extraction of total DNA from soil was used Power Soil DNA Kit, an aliquot of 5 µl of the extracted DNA was subjected to electrophoresis on agarose gel 1 % (w/v) stained with ethidium bromide (0,5 mg/ml gel) in TAE buffer. Was used as standard molecular 2 µl of Low Mass DNA Ladder. The gel was subjected to an electric field of 80 V for approximately 30 min and then photo-documented. The extracted DNA was also quantified by spectrophotometer. For purification of PCR products was used Kit GFXTM PCR DNA and Gel Band Purification by following the manufacturer's directions and visualized on agarose gels and subjected to an electric field of 80 V for approximately 30 min. With tags generated by using a primer specific 16S constructed a data matrix and calculated the diversity of the samples, using the Jaccard coefficient and UPGMA. The results were generated from the software FreeTree by which a dendrogram was obtained. The genotypes were grouped into three groups among the samples, group I: genotype accesses of the region 1, group II: genotype accesses of region 2, and group III: visualize genotype accesses of the region 3, checking the genetic distance between accesses collected from the regions. The high degree of polymorphism identified among samples represents heterozygosity for this marker, which can contribute to infer genetic relationships. Such diversity will in comparison studies of the diversity present in genomic databases. Thus, we conclude that there is diversity among the genotypes of the studied samples. The 16S rRNA gene has been used extensively in phylogenetic inference of microorganisms, however, because it is an initial study is still in the process of identification the total sequences of mangroves to continue with studies analyzing sequences isolated. In an attempt to attribute function to these genes and then use the potential of lineages. Observing this, we realize that the genotypes showed diversity among the samples collected. Such diversity will in comparison studies of the diversity present in genomic databases. Thus, we conclude that there is diversity among the genotypes of the studied samples. These results allow to conclude that the mangrove has exclusive characteristics, besides contributing to initial information on the study, to select genotypes that may be possible potential in bioremediation and other biotechnological products and industrial to be explored from the knowledge that diversity.
